# Discovery and Validation of a Compound to Target Ewing’s Sarcoma

**DOI:** 10.3390/pharmaceutics13101553

**Published:** 2021-09-24

**Authors:** Ellie Esfandiari Nazzaro, Fahad Y. Sabei, Walter K. Vogel, Mohamad Nazari, Katelyn S. Nicholson, Philip R. Gafken, Olena Taratula, Oleh Taratula, Monika A. Davare, Mark Leid

**Affiliations:** 1 Departments of Pharmaceutical Sciences, College of Pharmacy, Oregon State University, Corvallis, OR 97331, USA; ellie.esfandiari@gmail.com (E.E.N.); fsabei@jazanu.edu.sa (F.Y.S.); walter.vogel@oregonstate.edu (W.K.V.); nazzaromj@gmail.com (M.N.); olena.taratula@oregonstate.edu (O.T.); mark.leid@wsu.edu (M.L.); 2 Department of Pharmaceutics, College of Pharmacy, Jazan University, Jazan 88723, Saudi Arabia; 3 Division of Pediatric Hematology & Oncology, Department of Pediatrics, Oregon Health & Science University, Portland, OR 97239, USA; nickatel@ohsu.edu; 4 Proteomics & Metabolomics Shared Resource, Fred Hutchinson Cancer Research Center, Seattle, WA 98109, USA; pgafken@fredhutch.org; 5 Papé Pediatric Research Institute, Oregon Health & Science University, Portland, OR 97239, USA; 6 Department of Integrative Biosciences, Oregon Health & Science University, Portland, OR 97239, USA

**Keywords:** Ewing’s sarcoma, chemotherapy, cancer, ML111, drug discovery, nanoparticle drug delivery, high-throughput screening

## Abstract

Ewing’s sarcoma, characterized by pathognomonic t (11; 22) (q24; q12) and related chromosomal ETS family translocations, is a rare aggressive cancer of bone and soft tissue. Current protocols that include cytotoxic chemotherapeutic agents effectively treat localized disease; however, these aggressive therapies may result in treatment-related morbidities including second-site cancers in survivors. Moreover, the five-year survival rate in patients with relapsed, recurrent, or metastatic disease is less than 30%, despite intensive therapy with these cytotoxic agents. By using high-throughput phenotypic screening of small molecule libraries, we identified a previously uncharacterized compound (ML111) that inhibited in vitro proliferation of six established Ewing’s sarcoma cell lines with nanomolar potency. Proteomic studies show that ML111 treatment induced prometaphase arrest followed by rapid caspase-dependent apoptotic cell death in Ewing’s sarcoma cell lines. ML111, delivered via methoxypoly(ethylene glycol)-polycaprolactone copolymer nanoparticles, induced dose-dependent inhibition of Ewing’s sarcoma tumor growth in a murine xenograft model and invoked prometaphase arrest in vivo, consistent with in vitro data. These results suggest that ML111 represents a promising new drug lead for further preclinical studies and is a potential clinical development for the treatment of Ewing’s sarcoma.

## 1. Introduction

Ewing’s sarcoma, a small and round blue-cell malignancy arising from bone or soft tissue, most commonly affects children and adolescent patients. Ewing’s sarcoma is a rare aggressive cancer, posing substantial clinical challenges, and while prognoses have improved for young patients or patients with localized disease, outcomes for metastatic or relapsed disease remain dire [1,2]. Currently, standard treatment includes multimodal strategies with combinations of chemotherapy (vincristine, doxorubicin, cyclophosphamide, and ifosfamide), surgery, and radiation [3,4]. Targeted therapies for Ewing’s sarcoma have not advanced to the clinic despite precise knowledge of the etiology of the disease for nearly 30 years.

Up to 90% of Ewing’s sarcoma cases result from reciprocal chromosomal translocation involving the *EWSR1* (EWS) gene, which encodes an RNA-binding protein, and the gene encoding Friend leukemia virus integration 1 (FLI1), a member of the E26 transformation-specific (ETS) family of transcription factors, generating the pathognomonic EWS-FLI1 chimeric fusion protein. EWS-FLI1 and related fusion proteins contain the amino-terminus of EWSR1 harboring a strong transcriptional activation domain fused in frame with the carboxy terminus of FLI1, which contributes a highly promiscuous ETS-type DNA binding domain [5,6,7]. EWS-FLI1 and related fusion proteins regulate the expression of a large network of genes and dysregulation of this network underpins Ewing’s sarcoma pathogenesis, at least in part [8,9,10]. Notably, ectopic expression of EWS-FLI1 confers oncogenic and/or tumorigenic properties in permissive cell types [5,11,12].

EWS-FLI1 and related chimeric proteins are not expressed in untransformed cells; thus, Ewing’s sarcoma should be highly amenable to precision medicine-based approach [13]. However, currently there are no FDA-approved, molecularly targeted treatments for Ewing’s sarcoma. Efforts are underway to discover and validate pharmacological or other therapeutic approaches that directly downregulate EWS-FLI1 or target downstream or synthetic lethal vulnerabilities generated by this fusion oncoprotein. Agents being investigated in the relapsed Ewing’s sarcoma setting include epigenetic therapies (e.g., inhibitors of lysine-specific demethylase 1 (LSD1), histone deacetylases, and bromodomain-containing proteins), inhibitors of various downstream components of the EWS-FLI1 transcriptional network (TKI-216), agents that bind to DNA and disrupt processing of DNA by multiple pathways (e.g., plicamycin and trabectedin), CD99 targeting agents (clofarabine/cladribine and anti-CD99 antibodies), an anti-insulin-like growth factor receptor antibody (Ganitumab), and others [3]. Even if novel, molecularly targeted agents were to become available, the nearly inevitable development of therapeutic resistance to targeted agents necessitates a diverse or expanded pharmacological pipeline for potential second-line use.

Here, we describe results from a forward pharmacological screen that resulted in the identification of a compound, ML111, that potently inhibits the viability of Ewing’s sarcoma cells in vitro and in vivo. Chemical, biochemical, cell biological, and animal model data presented herein suggest that this small molecule has the potential to be an effective anti-tumor agent in the treatment of Ewing’s sarcoma.

## 2. Materials and Methods

### 2.1. Chemicals

2-amino-4-(3-methoxyphenyl)-4H-benzo[h]chromene-3-carbonitrile (ML111; PubChem SID 3323178) was purchased from ChemBridge (San Diego, CA, USA, #5307066) or synthesized according to the previous literature [14]. mPEG–PCL (methoxy poly(ethylene glycol)-b-poly(ε-caprolactone), MW: 5 k–10 k) was obtained from Advanced Polymer Materials Inc. (Montreal, QC, Canada), and SiNc was obtained (silicon 2,3-naphthalocyanine bis(trihexylsilyloxide)) from Sigma-Aldrich (Milwaukee, WI, USA).

### 2.2. Primary Antibodies

Antibodies to caspase 3 (#14220), CDC20 (#14866), cyclin B1 (#12231), GAPDH (#5174), histone H3 (#4499), phospho-Ser10-histone H3 (pH3^Ser10^, #53348), PARP (#9532), α-Tubulin (#2125, used for immunoblot analyses), and α-Tubulin (#3873, used for immunocytochemistry) were obtained from Cell Signaling (Danvers, MA, USA). Antibody to FLI1 (ab15289) was obtained from Abcam (Waltham, MA, USA).

### 2.3. Cell Lines and Cell Culture

Most Ewing’s sarcoma cell lines were kind gifts from the laboratory of Dr. Marc Ladanyi at Memorial Sloan Kettering Cancer Center. All other cell lines were acquired from ATCC (Manassas, VA, USA). SK-ES-1 and SK-OV-3 were cultured in McCoy’s 5A Medium (Corning, Glendale, AZ, USA). SK-N-MC, MDA-MB-231, and HEK293 cells were maintained in Minimum Essential Medium (Corning). HCC78, CHP100, A-673, TC-71 and TC-32, H3122, ES-2, and H460 cells were grown in RPMI 1640 (Thermo Fisher Scientific, Waltham, MA, USA). Human umbilical vein endothelial cells (HUVECs) were cultured in supplemented Medium 200, according to the manufacturer’s instructions. All cell lines were also maintained in 1% penicillin/streptomycin (Thermo Fisher Scientific, Waltham, MA, USA), 4 mM glutamine, and 10% Fetal Bovine Serum (FBS, R&D Systems, Minneapolis, MN, USA), except for SK-ES-1 cells, which were cultured in media containing 15% FBS, and the cells were maintained at 37 °C in a humidified incubator with 5% CO_2_.

### 2.4. High-Throughput Screening

SK-ES-1 cells were seeded at 1.0 × 10^4^ cells/well in 384-well tissue culture plates for HTS studies. After 24 h, compounds or controls were added by using a Sciclone ALH3000 Liquid Handler (Perkin Elmer, Waltham, MA, USA), and incubations were conducted for an additional 48 h. The primary screen was conducted using 10 µM compound, and all compounds were tested in triplicate. Follow-on studies using Ewing’s sarcoma and non-Ewing’s sarcoma cell lines were conducted similarly, with the exception that a HPD300 dispenser (HP Inc., Palo Alto, CA, USA) was used for liquid handling, and wells contained 0.5 × 10^4^ cells. Cell viability was measured using a luciferase-based reagent, CellTiter-Glo (Promega, Madison, WI, USA). Compounds that inhibited Ewing’s sarcoma cell growth by 85% were considered “hits”, and the growth inhibitory activity of these compounds was confirmed in secondary screens using a full range of compound concentrations. Dose–response curves were fit and analyzed using Prism 6.0 (GraphPad Software, San Diego, CA, USA). DMSO and bortezomib were used as negative and positive controls, respectively, and the Z-factor [15] for each screening plate (Z′) was in the range of 0.5–0.9, validating the high-throughput screening assay.

### 2.5. Imaging Caspase 3/7 Activation

SK-ES-1 cells were seeded at a density of 8000 cells per well in 96-well plate. After 20 h the cells were treated in triplicate with ML111 (50 nM), cabozantinib (250 nM), or vehicle control (0.05% DMSO). CellEvent Caspase-3/7 Green Detection Reagent (Thermo Fisher Scientific, Waltham, MA, USA), a fluorogenic substrate for activated caspase 3/7, was added to the same wells following the manufacturer’s protocol, and the cells were live imaged by using the Incucyte (Sartorius, Bohemia, NY, USA) imaging system with data capture set for every 30 min. The CellEvent reagent is intrinsically non-fluorescent in healthy cells because the DEVD peptide inhibits the ability of the fluorescent dye to bind DNA. The activation of caspase 3/7 in apoptotic cells results in cleavage of the DEVD peptide, enabling the dye to bind DNA and produce a green, fluorogenic response. Cells that activate caspase 3/7 emit green fluorescence and are identified as unique objects by the software. The relative number of cells that activate caspase 3/7 was quantified by using the built-in Incucyte image analysis tools. Imaging was suspended after 23 h.

### 2.6. Annexin V Staining

Cells were stained with FITC-Annexin V and propidium iodide by using a kit (product number V13242) from Thermo Fisher Scientific (Waltham, MA, USA), according to the manufacturer’s instructions. Briefly, SK-N-MC (2 × 10^6^) cells were cultured in a 25 cm^3^ flask overnight, after which cells were exposed to ML111 (100 nM) or vehicle control (0.1% DMSO) at predetermined time points. Cells were harvested, washed with cold Dulbecco’s phosphate buffered saline, and collected by centrifugation. Pelleted cells were resuspended in 100 μL of annexin binding buffer and stained with 5 µL of FITC-annexin V and 1 µL of propidium iodide for 15 min at room temperature and in the dark. Additional annexin binding buffer (400 µL) was added; samples were kept on ice before analysis by flow cytometry using a BD Accuri C6 flow cytometer (BD Biosciences, San Jose, CA, USA). Flow data were analyzed using FlowJo software (FlowJo LLC, Ashland, OR, USA).

### 2.7. Z-VAD-FMK Rescue Experiments

SK-N-MC cells, 1 × 10^4^ cells/well in a 96-well plate, were treated with the cell-permeable, irreversible pan-caspase inhibitor Z-VAD-FMK (50 μM; APExBIO, Houston, TX, USA), or vehicle (DMSO, final concentration of 0.1%) for 2 h prior to addition of ML111 (100 nM) or vehicle. The cells were treated with ML111 or vehicle for an additional 24 h. Cell viability was determined by using the Calcein AM assay (Corning, Glendale, AZ, USA), as described previously [16].

### 2.8. Immunoblot Analyses

Cells (2 × 10^6^) were dispersed on 10 cm plates and incubated overnight. The medium was removed, and the cells were incubated with freshly diluted vehicle or ML111 at various concentrations for various times. Cells were washed with ice-cold PBS (pH 7.4) and scraped into ice-cold PBS and centrifuged (800× *g*) for 5 min at 4 °C. Cell pellets were lysed in NP-40 buffer (150 mM NaCl, 50 mM Tris-HCl pH 8.0, and 1% NP40) containing a protease inhibitor cocktail, and protein concentrations in the lysates were determined by using the BCA assay (Thermo Fisher Scientific, Waltham, MA, USA). Lysates (20–30 µg of total protein) were combined with the sample buffer and boiled for 10 min prior to separation by polyacrylamide gel electrophoresis.

Gels were blotted to nitrocellulose membranes, which were then blocked by using an Odyssey PBS Blocking Buffer (LI-COR Biosciences, Lincoln, NE, USA) prior to incubation with primary antibodies, following the manufacturer’s instructions. Immunoreactive bands were detected by use of fluorescently tagged secondary antibodies: goat anti-rabbit IRDye 800 CW and IR Dye 680 CW goat anti-mouse (LI-COR and both at 1:5000 dilution). A LI-COR Odyssey scanner was used to visualize, quantify, and analyze the intensities of resultant fluorescent bands using ImageStudio (version 2.1, LI-COR Biosciences, Lincoln, NE, USA).

### 2.9. Histology, Immunohistochemistry, and Slide Evaluation

Tumor tissues were fixed for 2 h at 4 °C in fresh 4% paraformaldehyde (Electron Microscopy Sciences, Hatfield, PA, USA) in PBS. Fixed tumors were washed with ice-cold PBS and incubated in PBS overnight at 4 °C and then transferred to 20% sucrose in PBS for 24–48 h prior to embedding (Tissue-Tek O.C.T. Compound, Sakura Finetek, Torrance, CA, USA). Tissues were sectioned using Leica CM 1850 UV Cryostat (Leica Biosystems, Buffalo Grove, IL, USA). Sections (8–10 µm) were washed with PBS and endogenous peroxidase was inactivated by incubating the sections with 3% hydrogen peroxidase for 20 min prior to blocking in FBS. Sections were incubated with anti-pH3^Ser10^ antibody at 4 °C overnight. Sections were trice washed with PBS and incubated with a biotinylated secondary antibody (IDSTM003, ID Labs, London, ON, Canada) for 2 h, followed by incubation with avidin-horseradish peroxidase (HRP) (IDSTM003, ID Labs) for 20 min. The slides were stained with 3,3’-diaminobenzidine (DAB; Vector Laboratories, Burlingame, CA, USA), according to the manufacturer’s protocol. A counterstaining with hematoxylin (MilliporeSigma, Burlington, MA, USA) for 10 s was performed at the end of the staining. The sections were mounted by using Aquatex (MilliporeSigma, Burlington, MA, USA) and visualized using a Zeiss Axio Imager Z1 microscope. Qualitative and quantitative analyses of sections were evaluated by five different slides in a blinded fashion.

### 2.10. Immunocytochemistry

SK-N-MC cells were grown on glass coverslips (VWR, Radnor, PA, USA) in complete medium and treated with ML111 (25 nM) or DMSO (0.1%) for 24 h. Cells were fixed (ice-cold methanol), rehydrated with PBS, and blocked with 10% FBS (R&D Systems) and incubated with anti-pH3^Ser10^ and anti-α-Tubulin, each at a dilution of 1:500. The cells were counterstained with 4′,6-diamidino-2-phenylindole (DAPI, Thermo Fisher Scientific, Waltham, MA, USA) and visualized by using a Zeiss Axio Imager Z1 microscope (Carl Zeiss, White Plains, NY, USA) with appropriate filters and a 63× oil-immersion objective. Color images were digitally overlaid using the Zeiss AxioVs40 software v.4.8.2.0.

### 2.11. Microtubule Polymerization Assay

Cell-free tubulin polymerization assays were conducted according to the manufacturer’s instructions (BK006P; Cytoskeleton, Inc., Denver, CO, USA). Briefly, tubulin proteins (>99% purity) were suspended in G-PEM buffer (80 mM PIPES, pH 6.9, 2 mM MgCl_2_, 0.5 mM EGTA, 1 mM GTP) with or without paclitaxel (10 µM; Cytoskeleton, Inc., Denver, CO, USA), colchicine (10 µM; Sigma-Aldrich, St. Louis, MO, USA), and ML111 (0.1 and 1.0 µM) in a 96-well plate, and the absorbance was measured continuously at 340 nm for 60 min (Synergy multimode microplate reader, BioTek, Winooski, VT, USA).

### 2.12. Cell Cycle Analysis

SK-N-MC cells (0.9 × 10^6^) were plated in 6 cm dishes and incubated overnight. The medium was exchanged, and the cells were treated with 100 nM ML111 or vehicle (0.1% DMSO). Cells were harvested at the times specified for each experiment, washed with PBS, fixed in 70% ice-cold ethanol, and stored overnight at −20 °C. Fixed cells were washed in PBS and resuspended in staining solution, 0.1 mg/mL propidium iodide containing 0.1 mg/mL ribonuclease A, and incubated for 30 min prior to DNA content analysis using a Cytomics FC 500 flow cytometer (Beckman Coulter, Indianapolis, IN, USA) using instrument software CXP 2.2.

### 2.13. Effect of ML111 on SK-N-MC Cell Proteomics

#### 2.13.1. Sample Preparation

SK-N-MC cells were treated with 100 nM ML111 for 1.5–24 h or vehicle (0.1% DMSO). Treated cells were trice washed with ice-cold PBS to remove culture media and nonadherent cells. Adherent cells were collected in ice-cold PBS, pelleted at 1000× *g*, and frozen in liquid N_2_. Frozen cell pellets were thawed in 5 volumes of lysis buffer: 4% SDS, 50 mM HEPES, pH 8, 10 mM DTT, 1 mM EDTA, 0.1 mM PMSF, 1 µg/mL pepstatin A, 5 µg/mL leupeptin, and 10 µM E64; the resulting lysate was sonicated and incubated at 75 °C with shaking at 1400 rpm for 15 min and clarified by centrifugation. The SDS was then removed by ultrafiltration (Amicon Ultra-15, 3000 MWCO, MilliporeSigma, Burlington, MA, USA) and exchanged into Lys-C digestion buffer: 7.5 M urea, 20 mM HEPES, and pH 8 (final dilution factor 1:125,000). The resulting protein extracts were reduced with the addition of 10 mM TCEP and incubated for 1 h at 55 °C, then alkylated by the addition of 20 mM 2-chloroacetamide and incubated for 30 min at room temperature. Lys-C (Promega, Madison, WI, USA) was added at a ratio of 40:1 (extract: protease) and incubated for 4 h at 37 °C with mixing. After diluting urea to 1 M with 20 mM HEPES, pH 8, trypsin (Promega, Madison, WI, USA) was added at a ratio of 50:1 (extract: protease) and incubated for 15 h at 37 °C with mixing. Digests were acidified with TFA to pH < 2 and desalted on Oasis HLB 3CC cartridges (Waters, Milford, MA, USA). An aliquot of each digest (35 µg of protein) was labeled with one of six unique isobaric tags (TMTsixplex, #90061, Thermo Fisher Scientific, Waltham, MA, USA) in 35 mM triethylammonium bicarbonate, 29% acetonitrile, for 1 h at RT, then quenched with the addition of 0.27% hydroxlamine for 15 min at RT. The samples were acidified with TFA, combined, desalted on Oasis HLB 3CC cartridges, and dried by vacuum centrifugation.

#### 2.13.2. Basic Reverse-Phase Chromatography

The combined sample was resuspended in 200 µL total volume of 5/95% (*v*/*v*) acetonitrile/10 mM aqueous ammonium bicarbonate, pH 8 (buffer A), and loaded on to a Zorbax Extend-C18 column (2.1 × 150 mm, 5 µm particle size, p/n 773700-902, Agilent (Santa Clara, CA, USA) for basic reverse-phase fractionation. The sample was eluted from the column at a flowrate of 250 µL/min with a 1–40% gradient of 90/10% (*v*/*v*) acetonitrile/5 mM aqueous ammonium bicarbonate, pH 8 (buffer B) over 50 min, followed by 40–95% buffer B over 5 min, and then held at 95% buffer B for an additional 5 min. Eluting peptides were monitored by absorbance at 210 nm, and 250 µL fractions were collected and combined by concatenation into 20 pools. The pools were subsequently taken to near-dryness by vacuum centrifugation and brought up to 20 µL in 2/98% (*v*/*v*) acetonitrile/0.1% aqueous formic acid in preparation for LC-MS analysis.

#### 2.13.3. Mass Spectrometry

Sample pools were analyzed on an EASY-nLC 1000 nanoflow LC-coupled Orbitrap Fusion mass spectrometer (Thermo Fisher Scientific, San Jose, CA, USA). The LC system was configured with a PepMap RSLC EasySpray column (75 µm ID × 50 cm, 2 µm particle size, 100 Å pore size, Thermo Fisher Scientific, San Jose, CA, USA) and maintained at 40 °C during operation. Diluted peptide samples were loaded directly onto the column and eluted with a 2–5% gradient of acetonitrile in 0.1% aqueous formic acid from over 5 min, then to 30% over 180 min, then to 50% over 10 min, and held at 50% for 2 min followed by a 2 min gradient to 90% and held at 90% for 10 min, all at a flow rate of 300 nL/min. Eluting peptides were ionized by electrospray using an EASY-IC source operated at 2.1 kV in positive-ion mode. Data-dependent analysis (DDA) used the high-resolution Orbitrap mass analyzer for both MS (precursor ion scans) and MS/MS analysis (tandem mass scans).

MS spectra were recorded in the Orbitrap over a range of 350–1500 *m*/*z* at a resolution of 120,000 (fwhm, *m*/*z* 200) and AGC setting of 400,000 ions and a maximum injection time of 50 ms. Monoisotopic precursor ions (instrument MIPS program) of charge state 2–7 that met a minimum signal intensity of 5000 were selected for MS/MS analysis. Ions meeting these criteria, within a mass tolerance of 10 ppm, were then dynamically excluded from reanalysis for 30 s. The selected ions were isolated (1.2 *m*/*z*-wide window) by the quadrupole and subjected to higher-energy collisional dissociation (HCD) at a normalized collision energy of 40%. MS2 spectra, with first mass 100 *m*/*z*, were recorded in the Orbitrap mass analyzer at a resolution of 15,000 (fwhm, *m*/*z* 200) and AGC set to 50,000 ions for a maximum ion injection time of 160 ms. MS spectra were recorded in the Orbitrap over a range of 350–1500 *m*/*z* at a resolution of 120,000 (fwhm, *m*/*z* 200) and AGC setting of 400,000 ions and a maximum injection time of 50 ms. Monoisotopic precursor ions (instrument MIPS program) of charge state 2–7 that met the minimum signal intensity of 5000 were selected for MS/MS analysis. Ions meeting these criteria, within a mass tolerance of 10 ppm, were then dynamically excluded from reanalysis for 30 s. The selected ions were isolated (1.2 *m*/*z*-wide window) by the quadrupole and subjected to higher-energy collisional dissociation (HCD) at a normalized collision energy of 40%. MS2 spectra, with first mass 100 *m*/*z*, were recorded in the Orbitrap mass analyzer at a resolution of 15,000 (fwhm, *m*/*z* 200) and AGC set to 50,000 ions for a maximum ion injection time of 160 ms.

#### 2.13.4. Data Analysis and Protein Quantification

A Proteome Discoverer (version 2.2.0.388, Thermo Fisher Scientific, San Jose, CA, USA) was used to combine instrument raw data files and used to search a combined Uniprot Reference Sequence UP000005640 human protein database (26 May 2018, 73,112 entries) and the cRAP contaminant database (68 entries) [17] for matches relative to the tandem mass spectra by using Sequest HT [18]. The search parameters were set for cleavage by trypsin, allowing up to two missed cleavage sites. The mass tolerances were 10 ppm for precursor and 0.6 Da for fragment ions. Oxidation on methionine, carbamidomethylation on cysteine, acetylation of the protein amino terminus, and TMT-tag modification of lysine and amino terminus were permitted variable modifications. Percolator [19] was used to filter peptide–spectrum matches against a decoy database to assign a false discovery rate (FDR) to peptide–spectrum matches, and protein identifications required minimum (FDR < 1%) peptide–spectrum matches and a separately calculated protein-level FDR of <1%. Protein matches to the cRAP database were filtered out of the results. Reporter ions from unique and razor peptides were quantified from HCD MS2 scans by using an integration tolerance of 20 ppm with the most confident centroid setting.

### 2.14. Preparation of ML111-Based Nanoparticles (ML111-NP)

Nanoparticles loaded with ML111 were prepared via a modified solvent evaporating method [20]. Briefly, 1 mL of mPEG-PCL (40–160 mg) in acetone was added to an equal volume of ML111 (4.5 mg, also in acetone) followed by the addition of 2 mL of aqueous dextrose solution (5%). The mixture was stirred for 30 s to obtain a homogenous solution and was left overnight to allow evaporation of the organic solvent. The resulting solution was centrifuged at 5000 rpm for 5 min, and the supernatant was filtered through a 0.2 µm nylon filter in order to obtain a clear nanoparticle solution. For fluorescence visualization, the near-infrared (NIR) dye (silicon 2,3-naphthalocyanine bis(trihexylsilyloxide), SiNc, 0.2 mg/mL) was co-encapsulated within ML111-NP, as previously described [20]. Co-encapsulation with SiNc allowed evaluation of the body distribution of nanoparticles at predetermined time points using a Pearl Impulse Small Animal Imaging System (LI-COR Biosciences, Lincoln, NE, USA). Mice were euthanized after the completion of the study, and tissues were harvested for ex vivo imaging in order to evaluate the accumulation of the nanoparticles in individual organs and in the xenograft [20].

### 2.15. Characterization of ML111-Based Nanoparticles

#### 2.15.1. In Vivo Assessment of the Toxicity of ML111-NP

Swiss Webster mice (4–6 weeks old; Charles River Laboratories, Wilmington, MA, USA) were randomly separated into three groups five mice receiving ML111-NP, empty-mPEG-PCL-NP, and 5% dextrose, respectively. Intravenous injections (150–200 µL volume) were based on body weight in order to ensure identical doses of polymer (680 mg/kg of mPEG-PCL) and ML111 (15 mg/kg) to all mice. Mice were dosed three times per week for three weeks. After treatment and euthanasia, blood samples were collected and submitted for complete blood chemistry analyses (IDEXX Veterinary Services; Portland, OR, USA). Serum biochemistry evaluations addressed the following organ toxicities: kidney, blood urea nitrogen level, and creatinine; heart and creatine kinase; and liver, alanine transaminase, aspartate transaminase, and alkaline phosphate. Toxicities affecting the blood were assessed by conducting complete blood counts and levels of red blood cells, white blood cells, hemoglobin, hematocrit, mean corpuscular volume, mean corpuscular hemoglobin, and mean corpuscular hemoglobin concentration. All determinations were compared across treatment groups by one-way ANOVA.

#### 2.15.2. Physical Parameters

The size, polydispersity index (PDI), and zeta potential (ζ) of nanoparticle-based preparations of ML111 were characterized by Dynamic Light Scattering (DLS; Malvern ZetaSizer Nanoseries, Malvern, UK), according to manufacturer’s instructions. Values represent mean of triplicate measurements of each parameter. The morphology of ML111-NP nanoparticles was evaluated with cryogenic transmission electron microscopy (cryo-TEM) using a 400-mesh copper grid. Images were recorded with a K2 Summit camera (Gatan Inc., Pleasanton, CA, USA) in counting mode on a Talos Arctica microscope (Thermo Fisher Scientific, Waltham, MA, USA) that was operated at 200 kV. Cryo-TEM images were collected with a defocus range of 2–4 µm.

The amount of ML111 loaded in mPEG-PCL-based nanoparticles was quantified by reverse-phase liquid chromatography by using a Shimadzu HPLC system configured with a Zorbax C18 column (4.6 × 75 mm, 3.5 µm particle size, p/n 866953-902, Agilent (Santa Clara, CA, USA) after developing a standard curve with known amounts of ML111. Unknown samples were eluted from the C18 column at a flow rate at 0.3 mL/min by using an isocratic method with a mobile phase consisting of 60% (*v*/*v*) acetonitrile in water containing 0.1% TFA. ML111 was detected at 228 nm with a retention time of 6.94 min. Encapsulation efficacy was calculated as the amount of ML111 loaded in nanoparticle as a ratio of the initial amount of ML111 in the encapsulation procedure, and the loading capacity was calculated as the percentage of ML111 in nanoparticles in relation to the total mass of nanoparticles.

#### 2.15.3. In Vitro ML111 Release

In vitro ML111 release was assessed using a modified equilibrium dialysis method [21]. Freshly prepared ML111-NP (2.5 mL of 2 mg/mL solution of ML111) was placed in a Slide-A-Lyzer G2 dialysis cassette (20,000 MWCO, Thermo Fisher Scientific, Waltham, MA, USA) and dialyzed against the phosphate buffer saline containing 0.1% Tween 20 (PBST) at pH 7.4 and 37 °C. Samples (50 µL) were withdrawn at pre-determined times (0–144 h) and replaced with a 50 µL volume of a fresh buffer. Samples were quantified as described above. The release of ML111 from nanoparticles was fit to the following equation:

$$\frac{M_{t}}{M_{\infty }}=M_{max}-A_{1}e^{\left(\frac{t}{-\tau 1}\right)}-A_{2}e^{\left(\frac{t}{-\tau 2}\right)}$$

where *M_t_* is the cumulative release of ML111 at time *t*; *M*_∞_ is the cumulative release of ML111 at infinite time; *A*_1_ and *A*_2_ are the relative amounts of ML111 release associated with *τ*1 and *τ*2, the mean lifetimes of the fast-release and slow-release phases, respectively; and *M_max_* is the total amount of ML111 release from both phases. The parameters were estimated by nonlinear least squares procedures using Marquardt’s algorithm [22] and are reported ±95% confidence interval.

#### 2.15.4. Cellular Toxicity Assay of ML111-NP

The cellular toxicity of empty-NP (mPEG-PCL) and ML111-NP was assessed in SK-N-MC and HEK239 cells using the Calcein AM cell viability assay (Corning, Glendale, AZ, USA). The cells were seeded in 96-well flat bottom plates at a density of 1 × 10^4^ cells per well. The cells were treated with empty-NP (mPEG-PCL; 0.39–400 µg/mL) and ML111-NP formulation (0.95–500 nM) for 48 h. The cells were then incubated with 10 µM Calcein AM in DPBS buffer, as previously described [23].

#### 2.15.5. Cellular Internalization of ML111-NP

Cells numbering 2.5 × 10^5^ SK-N-MC cells/well were plated in 6-well plates and permitted to attach overnight prior to incubation with ML111-NP co-encapsulated with a SiNc dye (20 µg/mL) for 24 h. Cells were co-stained with DAPI (NucBlue Live Cell Stain, Thermo Fisher Scientific, Waltham, MA, USA) and imaged using a Keyence BZ-X fluorescence microscope equipped with a Cy7 filter cube for SiNc (excitation, 710 nm; emission 775 nm) and DAPI (excitation, 360 nm; emission, 460 nm).

### 2.16. In Vivo Efficacy of ML111-Based Nanoparticles (ML111-NP)

A mouse xenograft model of Ewing’s sarcoma was created using athymic nu/nu mice (4–6 weeks old; Charles River Laboratories, Wilmington, MA, USA). Mice were injected subcutaneously into the right flank with 2.5 × 10^6^ SK-N-MC cells in 100 µL of MEM medium prepared in Matrigel matrix (1:1 ratio, Corning Life Science). When tumors reached a size of 40–60 mm^3^, mice were randomly separated into four groups (five mice/group), as described above. Two doses of ML111-NP were tested, 4.5 and 15 mg/kg. Tumor diameter measurements were made by using a caliper every other day for 28 days. Tumor volume was calculated by (width^2^ × length) × 0.5.

### 2.17. Statistical Analysis

Statistical significance was calculated using a Student’s *t*-test and paired control and test samples were analyzed with Prism 6.0 software (GraphPad Software, San Diego, CA, USA).

## 3. Results

### 3.1. Discovery of Lead Compound Using High-Throughput Functional Screening

**Figure 1:**
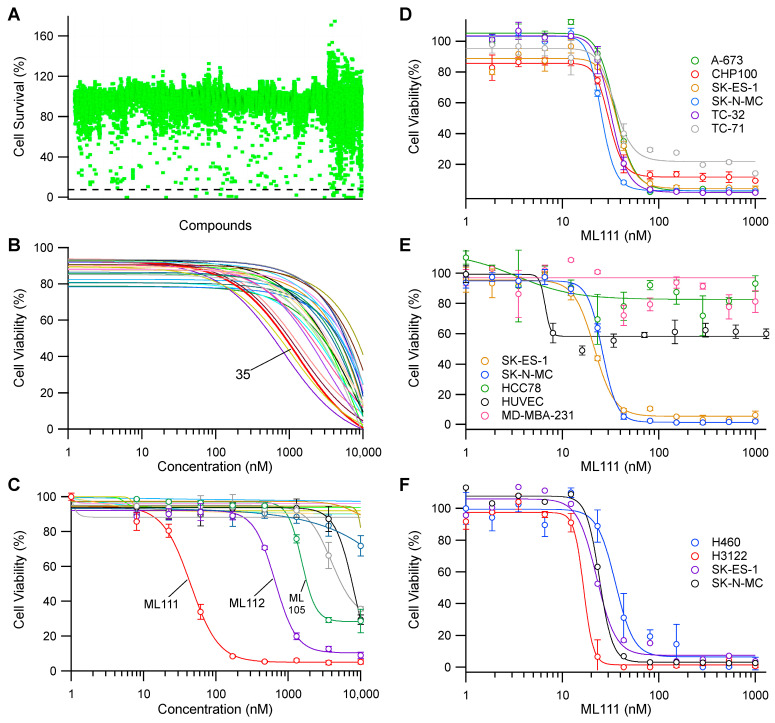
Lead compound selection process and the results of each step. (**A**) Primary screen: A scatter plot of the percentage cell survival of SK-ES-1 treated with 10,500 pure compounds at 10 μM for 48 h. Each dot represents one compound. Fifty-two hits (0.5% of the screened chemicals) were selected for further studies based on their ability to reduce cell viability by 85% (the dashed line shows the cutoff for selection of hits). (**B**) Secondary screen: Confirming the activity of the identified hits from the primary screen. The dose–response curve studies confirmed the activity of twenty-seven (51.9%) compounds. Compound 35 was selected for the tertiary screen. (**C**) Tertiary screen: The dose–response analysis of compound 35 and its structural analogs. ML111 was selected as the lead compound for further studies. (**D**) Dose–response analysis of Ewing’s sarcoma cell lines with either type I or type II fusion protein after ML111 treatment. (**E**,**F**) Effect of ML111 on various cancerous and primary cell lines. Data in (**C**–**F**) shown as mean ± SD (*n* = 3).

**Scheme 1:**
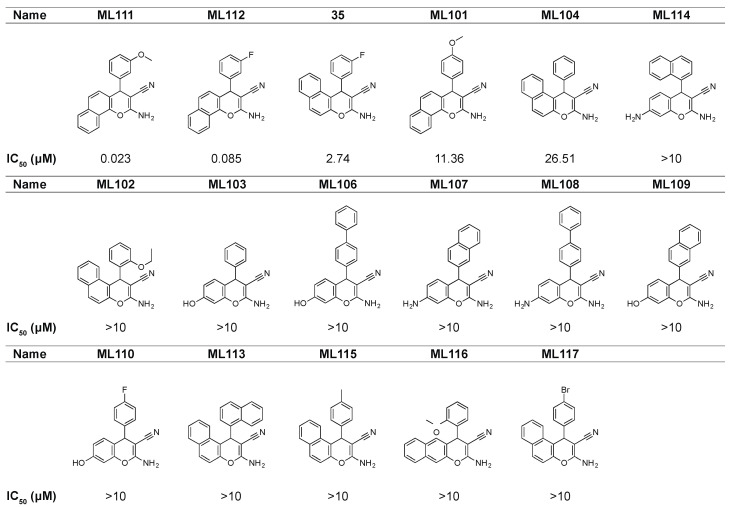
Potency of ML111 analogs. Scheme shows the structures and SK-ES-1 cell growth inhibitory activity as dose–response IC_50_ values.

In order to identify new agents that inhibit the viability of Ewing’s sarcoma cells, we conducted a phenotypic, high-throughput screen (HTS) using SK-ES-1 cells, a well characterized Ewing’s sarcoma cell line harboring a type II EWS-FLI1 oncofusion protein [24,25] and a chemical library composed of small molecules from the ChemBridge DIVERSet-CL and CORE Libraries (https://www.chembridge.com/screening_libraries (accessed July 2016)). SK-ES-1 cells were treated individually with 10,500 compounds in the primary screen, each at a single concentration of 10 μM for 48 h, and cellular viability was assessed using Cell-Titer Glo (Figure 1A). Cells were treated with bortezomib and vehicle (0.1% DMSO) as positive and negative controls, respectively. Positive hits were defined as compounds that inhibited SK-ES-1 cell viability by at least 85%. A total of 52 compounds were selected for subsequent dose–response studies. The activity of 27 of these compounds was confirmed, but the majority exhibited low potency (IC_50_ > 1 µM; Figure 1B). However, five compounds segregated from the rest, and among these five compounds, Compound 35 (Figure 1B) was selected for further analysis because of its relative drug-like structure, according to Lipinski’s rules [26] and ease of synthesis. In silico screening of an additional ~240,000 library compounds identified 16 new compounds that exhibited greater than 80% structural similarity to Compound 35 (Scheme 1). Among the latter analogs, a compound now named ML111 exhibited ~20-fold higher potency compared to Compound 35 and, thus, was selected for further studies (Figure 1C).

### 3.2. ML111 Inhibits Viability of Ewing’s Sarcoma Cell Lines Harboring Both Type I and II Fusion Proteins

The inhibitory effect of ML111 was tested against an expanded panel of established Ewing’s sarcoma cells harboring both type I and II EWS-FLI1 fusion proteins. All tested Ewing’s sarcoma cell lines exhibited comparable sensitivity to ML111 (Figure 1D and Table S1), indicating that the effect of ML111 on Ewing’s sarcoma viability was independent of the type of EWS-FLI1 fusion protein expressed.

We then performed cell viability studies on a panel of non-Ewing’s sarcoma cancer cell lines to assess selectivity and determine if the inhibitory effect of ML111 is generalizable to cancers representing diverse histologies. ML111 inhibited growth of the ovarian cancer cell line ES-2 and lung cancer cell line H460 with potencies similar to that of the Ewing’s sarcoma cell lines (Figure 1E and Table S1). However, the breast cancer cell line MDA-MB-231, non-small cell lung carcinoma (NSCLC) cell line HCC78, and ovarian cell line SK-OV-3 were largely resistant to ML111 (Figure 1E and Table S1). An additional NSCLC line, H3122 (EML4-ALK), exhibited enhanced sensitivity to ML111 relative to Ewing’s sarcoma cells. Non-cancerous human embryonic kidney cells 293 (HEK293) (Table S1) and primary human umbilical vein endothelial cells (HUVEC) were relatively insensitive to ML111 (Figure 1E). Overall, these data suggest that ML111 is a promising anti-cancer agent for treatment of Ewing’s sarcoma and possibly specific molecular subset(s) of cancers.

### 3.3. Single Enantiomer of ML111 Responsible for Anti-Viability Effect in Ewing’s Sarcoma Cells

**Figure 2:**
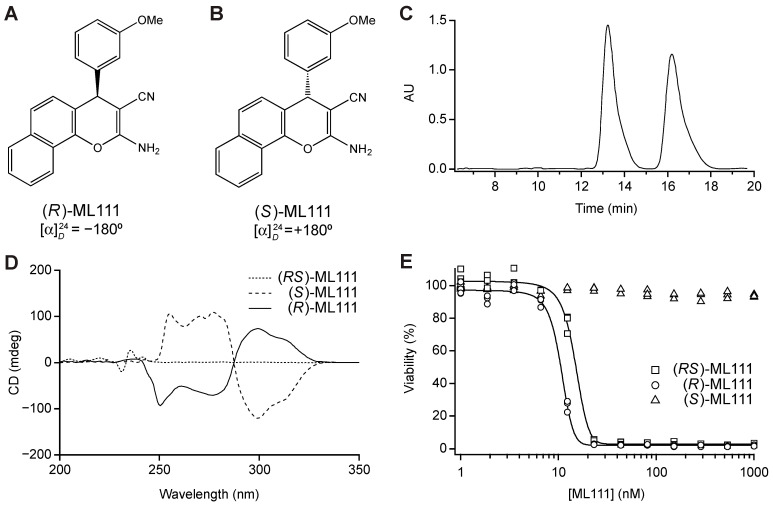
Enantiospecificity of ML111 action in Ewing’s sarcoma cells. (**A**,**B**) (*R*)-ML111 and (*S*)-ML111 chemical structures. (**C**) The racemate ML111 was resolved into its individual enantiomers by chiral chromatography. HPLC analysis of racemate clearly identified two single enantiomer peaks. AU: absorbance units. (**D**) CD spectra of (*R*)-ML111, (*S*)-ML111, and racemate ML111. The first enantiomer to elute from the column in (**C**) shows a specific rotation value of −180°, while that for the second enantiomer is +180°. (**E**) Dose–response analysis of racemate ML111, (*S*)-ML111, and (*R*)-ML111 in SK-N-MC cells with various concentrations of each compound ranging from 0.001 to 1 μM for 48 h.

ML111 harbors one chiral center (Figure 2A,B) and racemic ML111 was resolved into its individual enantiomers by chiral HPLC in order to determine the enantiospecificity of the compound. The analytical trace is consistent with the presence of two well-separated enantiomers with retention times of 13.3 and 16.5 min (Figure 2C). The specific rotation of individual enantiomers was determined by polarimetric analysis. Circular dichroism spectroscopic analyses of the isolated enantiomers demonstrated the expected mirror image spectra, while the racemate had zero net rotation of polarized light, indicating a mixture of two isomers in equal proportions (Figure 2D). The relative anti-viability potencies of (*RS*)-ML111, (*S*)-ML111, and (*R*)-ML111 were determined using SK-N-MC cells in vitro. (*R*)-ML111 was found to be the active enantiomer with an IC_50_ of 16.5 ± 1.3 nM (IC_50_ of racemic ML111 was 19.8 ± 3.2 nM, Figure 2E). (*S*)-ML111 was devoid of activity in these studies (Figure 2E). These results demonstrate enantiomeric-specific activity of ML111.

### 3.4. ML111 Induces Caspase-3/7-Dependent Apoptosis

**Figure 3:**
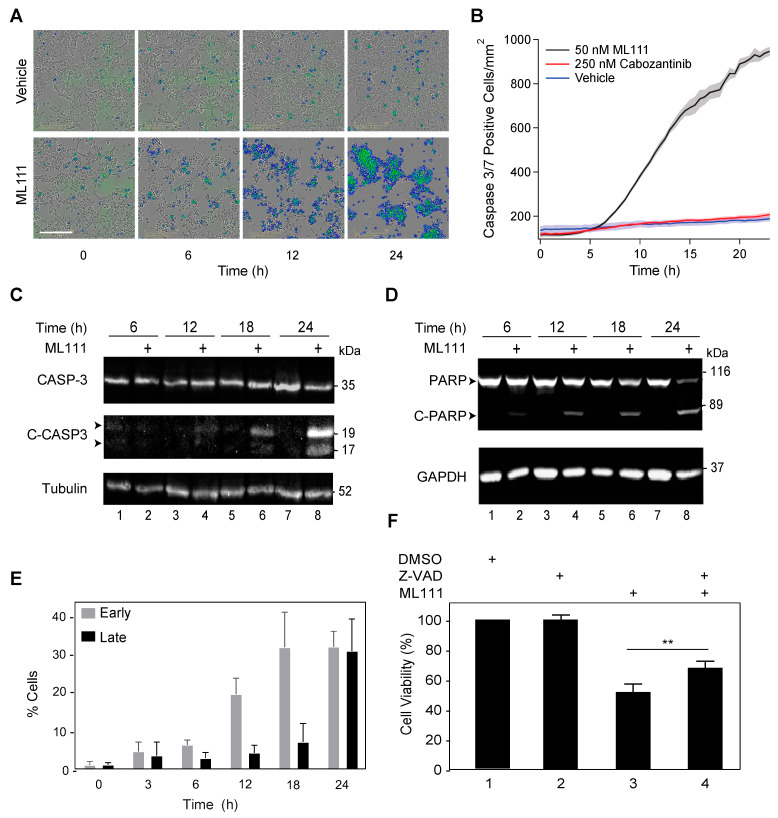
Induction of caspase-dependent apoptosis by ML111 in a time-dependent manner. (**A**) Induction of caspase 3/7 activity after vehicle, ML111 (50 nM), or cabozantinib (250 nM) treatment at indicated time intervals. Green nuclear fluorescence indicates cellular caspase 3/7 activation as detected by using CellEvent Caspase-3/7 Green Detection Reagent. (**B**) Quantification of caspase-3/7 activated cells at indicated times. The mean is shown, and the shaded regions represent the SEM (*n* = 4). (**C**) Immunoblot analysis of pro-caspase 3 cleavage forming active caspase 3 after 12 h of treatment with ML111. (**D**) Western blot analysis of PARP cleavage as a marker of caspase 3 activation and apoptosis. (**E**) Measurement of early and late apoptosis in ML111 treated cells as measured by annexin V and propidium iodide staining and flow cytometry. (**F**) Caspase inhibitor Z-VAD attenuates ML111-mediated induction of apoptosis. Asterisks (**) indicate statistically significant rescue of ML111 induced apoptosis by Z-VAD treatment (*p* < 0.01). GAPDH and α-tubulin were used as loading controls. CASP-3: Caspase 3; C-CASP3: Cleaved Caspase 3; C-PARP: Cleaved PARP. Scale bar in panel A = 200 µm.

We performed live-cell imaging to determine if the loss of cell viability observed in ML111-treated Ewing’s sarcoma cells is due to cytostatic or cytotoxic/apoptotic mechanisms. For these experiments, SK-ES-1 cells were treated with ML111 followed by the addition of CellEvent™ Caspase-3/7 detection reagent and imaged using the Incucyte Live Cell Analysis System. An increase in caspase 3/7-activated green fluorescent cells (outlined in blue line) is observed as early as 6 h after ML111 treatment (Figure 3A). To assess the specificity of ML111 (50 nM) in caspase activation and to ensure that the cells are not generically hypersensitive to perturbation of other signaling pathways, we tested cabozantinib (250 nM), a tyrosine kinase inhibitor (TKI) with multiple targets. Quantification of green fluorescent cells over the course of imaging shows 5-fold greater activation of caspase 3/7 in ML111-treated cells (946.8 ± 20.3) at 24 h, as compared to vehicle (189.4 ± 12.1) or cabozantinib treated cells (207.4 ± 11.1) (Figure 3B). Caspase 3/7 activation after ML111 treatment is reproducible in CHP100, A673, and TC-32 Ewing’s sarcoma cell lines (Figure S1); dasatinib, another multikinase inhibitor with a broad-spectrum kinase-inhibition profile, was used in the latter studies to ascertain selectivity of ML111 in inducing apoptosis.

Caspases, a family of cysteine endoproteases, play a central role in all known pathways of apoptosis [27]. We examined caspase 3/7 cleavage by immunoblot analyses to validate enhanced caspase 3/7 activity observed in live-cell studies (Figure 3A). These data reveal a time-dependent increase in cleaved caspase 3/7, albeit at the earlier hours (6 and 12 h); the levels are at or below the threshold of antibody detection since the events are occurring in a smaller cell populations. (Figure 3C). We confirmed these results using a second apoptosis marker, poly ADP-ribose polymerase (PARP) [28,29]. PARP cleavage also increases in a time-dependent manner in cells treated with ML111. (Figure 3D). Immunoblotting results showing increase in cleaved caspase 3/7 and cleaved PARP in ML111 treated cells are consistent with live-cell imaging studies (Figure 3A).

Next, we examined the localization of annexin V on the surface of SK-N-MC cells treated with ML111 by staining with an anti-annexin V antibody and flow cytometry. Untreated SK-N-MC cells exhibited very low levels of annexin V staining on the cell surface (Figure 3E, time 0). SK-N-MC cells treated with ML111 for at least 12 h exhibited robust Annexin V staining on the cell surface, indicating initiation of the apoptosis program. Similarly, co-staining these cells with propidium iodide (PI), a marker of cell death, shows that, after 24 h of ML111 treatment, a large number of cells are terminally apoptotic (Figure 3E).

Live-cell imaging studies indicated rapid and robust activation of caspase 3 and/or caspase 7 in SK-ES-1 and SK-N-MC cells treated with ML111, and all supporting data were consistent with a role for caspases in ML111-induced loss of viability (Figure 3B–D). Consistently, we found that the cell-permeant, irreversible pan-caspase inhibitor Z-VAD-FMK (Z-VAD) partially rescued cell viability of SK-N-MC treated with ML111 (Figure 3F), providing direct evidence for the role of caspase activation in the mechanism of ML111 action. Taken together, the above results conclusively demonstrate that ML111 induces apoptotic cell death in these Ewing’s sarcoma model cell lines.

### 3.5. ML111 Induces Prometaphase Arrest in SK-N-MC Cells Independent of an Effect on Microtubule Dynamics

**Figure 4:**
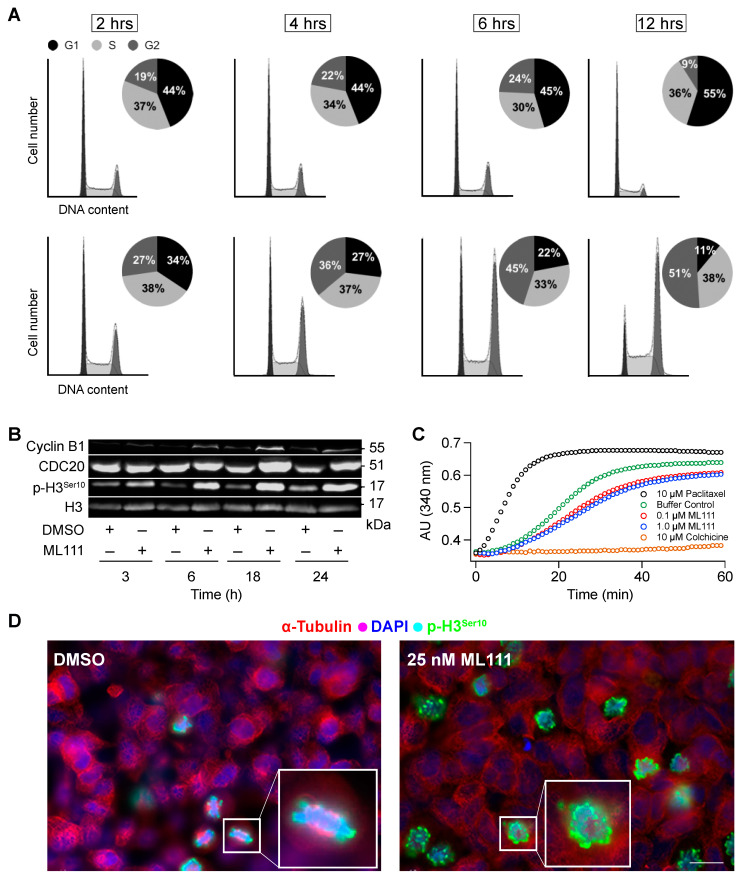
ML111 induced prometaphase arrest without altering microtubule dynamics. (**A**) Cell cycle analyses by flow cytometry in asynchronous SK-N-MC cells treated with 0.1% DMSO (upper panel) or 100 nM ML111 (lower panel). (**B**) Immunoblot analysis for cyclin B1, p-H3^Ser10^, and CDC20 in lysates from asynchronized SK-N-MC cells treated with 100 nM ML111 or 0.1% DMSO. Total histone H3 (H3) antibody was used as a control. (**C**) Cell-free tubulin polymerization assay. ML111 neither stimulated nor inhibited tubulin polymerization in vitro. Paclitaxel and colchicine were used as tubulin polymerization inducer and inhibitor agents, respectively. General tubulin buffer (Cytoskeleton, Inc.) was used as a control. AU: absorbance units. (**D**) ML111 induced prometaphase arrest. Asynchronized SK-N-MC cells were treated with either DMSO (0.1%) or ML111 (25 nM) for 12 h. The nuclei were stained with DAPI (blue), microtubules (α-tubulin, red), and mitotic chromosomes (p-H3^Ser10^, green). One representative result was shown from three independent experiments. Magnification: 63×. Scale bar = 10 μm.

The effect of ML111 on cell cycle dynamics was assessed in SK-N-MC cells to determine if ML111-induced cell death was associated with altered control of cell cycle. Treatment of asynchronous SK-N-MC cells with ML111 resulted in the accumulation of cells in the G2/M phase in a time-dependent manner (from 27% at 2 h to 51% at 12 h; Figure 4A, bottom panels), indicating the induction of cell cycle arrest at or near the G2/M checkpoint. We next analyzed the expression of three M-phase specific markers to confirm arrest in M phase: cyclin B1, phospho-Ser10-histone H3 (p-H3^Ser10^), and the cell division cycle 20 protein (CDC20). Degradation of cyclin B1 and CDC20 is required for cell cycle progression through the G2/M checkpoint [30]. Histone H3 is phosphorylated at Ser10 in late G2/M in association with mitotic chromatin condensation [31,32,33]. Treatment of SK-N-MC cells with ML111 resulted in the time-dependent accumulation of all three markers (Figure 4B), suggesting that ML111-induced M phase arrest.

Cell cycle arrest during M phase can be a result of disruption of microtubule polymerization. The effect of ML111 on microtubule polymerization was evaluated by using purified components in a cell-free assay. Unlike known microtubule regulators paclitaxel and colchicine, which strongly stimulated and inhibited microtubule polymerization, respectively, ML111 neither promoted nor inhibited microtubule polymerization (Figure 4C), suggesting that the induction of M phase arrest by ML111 is independent of its ability to affect microtubule polymerization directly.

We conducted immunocytochemical staining to define the point of ML111-induced arrest in SK-N-MC cells with more precision. Immunocytochemical staining with anti-p-H3^Ser10^, a marker of mitotic condensed chromatids, and counterstaining with DAPI revealed that asynchronous SK-N-MC cells treated with ML111 exhibited condensed but unaligned chromosomes, as compared to vehicle-treated cells (Figure 4D, right panel). This finding suggests that ML111 arrested SK-N-MC cells at prometaphase [34].

### 3.6. Quantitative Proteomic Analysis of ML111-Treated Ewing’s Sarcoma Cells

**Figure 5:**
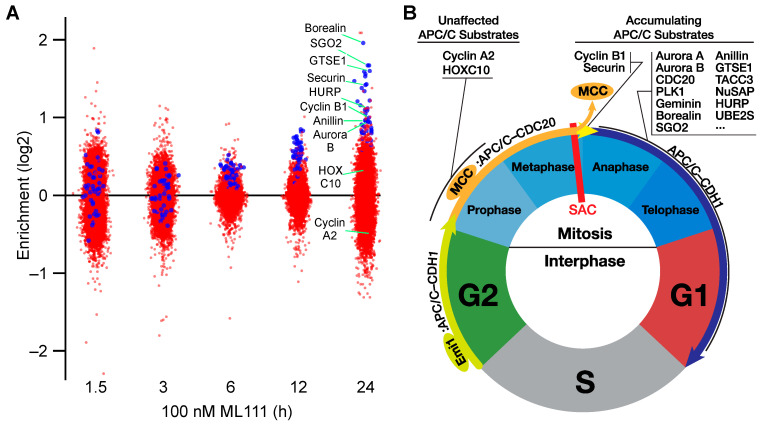
ML111-induced accumulation of APC/C substrates in SK-N-MC cells. (**A**) Scatter plot showing relative quantitative enrichment of 7122 proteins by mass spectrometry in response to ML111 relative to vehicle control in SK-N-MC cells. Known cell cycle proteins upregulated > 1.5-fold (log2 > 0.58) are shown in blue. (**B**) Diagram showing where the accumulating APC/C substrates would normally be ubiquitinated by the APC/C complex in the cell cycle. The diagram illustrates the dependence the cell cycle progression on APC/C co-activators CDC20 and CDH1, the spindle assembly checkpoint (SAC), mitotic checkpoint complex (MCC), and the location in the cell cycle where various substrates are expected to be ubiquitinated.

The effect of ML111 treatment on global cellular processes in SK-N-MC cells was assessed using an isobaric-tag peptide labeling approach to quantify changes in the whole cellular proteome. A total of 7471 non-redundant proteins were identified at a 1% FDR, of which 7122 were quantified across five treatment times, between 1.5 and 24 h (Table S2). Our analyses revealed that treatment of SK-N-MC cells with ML111 resulted in time-dependent stabilization of numerous cell cycle proteins, most of which are substrates for the anaphase-promoting complex/cyclosome (APC/C), an E3 ubiquitin ligase that targets mitotic factors for rapid proteasomal degradation (Figure 5A and Appendix A). APC/C activity drives cell cycle progression through mitosis and is the focus of the spindle assembly checkpoint (SAC), which blocks APC/C-mediated protein degradation and prevents premature chromosome separation (Figure 5B). ML111 treatment resulted in the accumulation of cyclin B1 and securin, which are normally degraded rapidly at the onset of mitotic exit, as well as the numerous other factors that are degraded in late mitosis and not required during interphase. However, not all substrates of APC/C were observed to accumulate in Ewing’s sarcoma cells treated with ML111. Two factors including cyclin A2 [35] and HOXC10 [36], which circumvent MCC inhibition of APC/C-CDC20-mediated ubiquitination and are normally degraded during late prophase or early prometaphase, did not accumulate in cells treated with ML111. NEK2A is also a known prophase APC/C substrate [37], but this protein was not detected in these studies. While we have no direct evidence for ubiquitination of cyclin A2 or HOXC10, these data suggest that the APC/C complex may be enzymatically capable of carrying out the E3 ligase reaction—at least using these factors as a substrate—suggesting that ML111 may not function as a direct or global inhibitor of APC/C in SK-N-MC cells.

### 3.7. Formulation of Nanoparticle-Based Drug Delivery System for ML111

**Figure 6:**
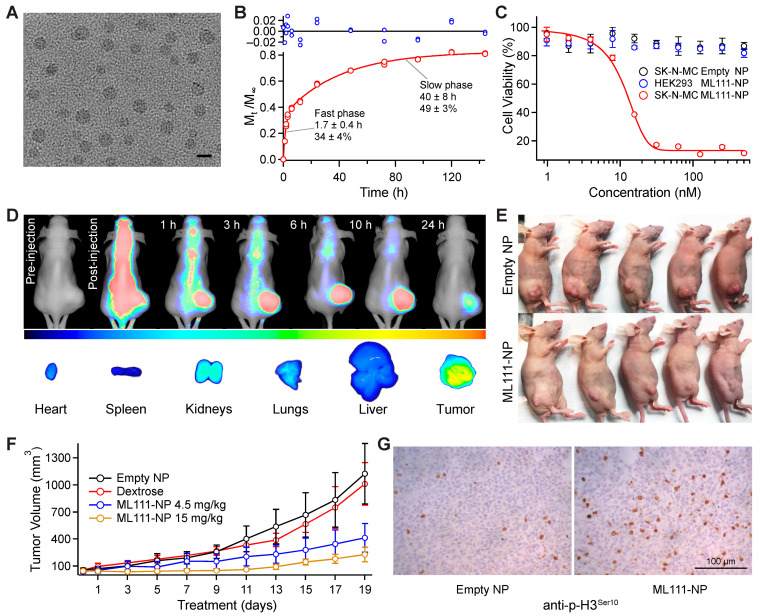
Characterization of ML111-NP. (**A**) Cryo-TEM image of ML111-NP. See Figure S2 for a full-resolution image. (**B**) ML111 released from nanoparticles in two phases in vitro. (**C**) Effect of empty NP and ML111-NP on viability of SK-N-MC and HEK293 cells after 48 h of incubation. (**D**) Fluorescence images of nude mice with subcutaneous SK-N-MC tumors pre-, post-, and at 1, 3, 6, 10, and 24 h after i.v. injection of ML111-NP (co-encapsulated with the NIR SiNc dye for fluorescence visualization) and fluorescence images of various organs and tumors at 24 h post-injection. Images were recorded on the Pearl Impulse Scheme 800 nm channel. (**E**) Representative mice bearing subcutaneous SK-N-MC xenografts and treated with empty nanoparticles or those containing ML111, as indicated. Mice are shown at necropsy. Reduction in xenograft tumor volume was observed in mice treated with ML111-NP. (**F**) A tumor growth profile after i.v. injections with ML111-NP, empty nanoparticles, or 5% dextrose. Data are shown as mean ± SD (*n* = 3). (**G**) Immunohistochemical staining with anti-p-H3^Ser10^ in SK-N-MC xenograft tissues from mice treated with empty nanoparticles or those loaded with ML111 (15 mg of ML111/kg). See Figure S6 for a quantification of the results. Scale bar = 20 nm in panel A and 100 µm in panel G.

A nanoparticle-based drug delivery system was used for in vivo efficacy studies of ML111 to overcome the limited aqueous solubility of the compound. For this purpose, we used biocompatible methoxy polyethylene glycol-poly(caprolactone) block polymers (mPEG-PCL). The use of the biodegradable mPEG-PCL-based nanoparticles allowed us to achieve high ML111 loading capacity due to the lipophilic PCL core yet maintain high water-solubility owing to the presence of the hydrophilic PEG shell. Various concentrations of mPEG-PCL (20–80 mg/mL in acetone) were mixed with a fixed concentration of ML111 (2.25 mg/mL, also in acetone) to optimize drug loading and stability in physiological solution. Use of 80 mg/mL of mPEG-PCL and 2.25 mg/mL of ML111 provided an encapsulation efficacy and loading capacity of 89.3% and 2.44%, respectively (Table S3). Cryogenic transmission electron microscopy images revealed spherical morphology for ML111-NP, with an average size of 21.1 ± 0.2 nm (Figure 6A and Figure S2). ML111-NPs have a mean hydrodynamic diameter of 31.8 ± 0.2 nm by dynamic light scattering (Figure S3 and Table S3) and a neutral zeta potential of 0.12 ± 0.01 (Table S3). The polydispersity index (PDI) of ML111-NP was less than 0.1 with a unimodal distribution (Table S3) [38]. These analyses demonstrated that the nanoparticle-based formulation of ML111 possesses the required size and a nearly neutral charge, both of which should result in extended blood levels, enhanced tumoral accumulation via passive targeting, minimal renal clearance, and impaired detection by macrophages [38]. The enhancement of solubility of ML111 in the mPEG-PCL nanoparticle allowed us to achieve a therapeutically relevant dosage (4.5–15 mg/kg) for in vivo assessment.

The in vitro release behavior of ML111-NP in the physiological buffer followed a typical biphasic pattern with a fast initial phase, during which ~34% of the ML111 cargo was released with a mean lifetime of 1.7 ± 0.4 h and a slow phase during which ~49% of ML111 was released with a mean lifetime of 40 ± 8 h (Figure 6B). The release of the remaining ~17% of the ML111 was too slow to be quantified. Collectively, these findings suggest that the spontaneous leakage of ML111 from this nanoparticle formulation is unlikely, and the preparation appears to exhibit inherent sustained release properties [39,40].

### 3.8. Effect of ML111-NP on Viability of SK-N-MC and HEK293 Cells

ML111 was co-encapsulated with a near-infrared (NIR) dye, SiNc, to assess intracellular uptake of ML111 nanoparticle formulation. The fluorescence signal of SiNc indicated substantial internalization of ML111-NP within SK-N-MC cells (Figure S4). ML111-NP, but not empty nanoparticles, inhibited the viability of SK-N-MC cells in vitro with an IC_50_ of 10.8 nM ML111 (Figure 6C). In contrast, ML111-NP did not inhibit the viability of HEK293 cells (Figure 6C), consistent with our earlier studies in non-Ewing’s sarcoma cell lines (Figure 1C). These findings also indicate that mPEG-PCL formulation is nontoxic to non-malignant HEK293 cells.

### 3.9. ML111 Decreases Tumor Growth in a Mouse Xenograft Model of Ewing’s Sarcoma

A mouse model was employed to evaluate the in vivo efficacy of ML111-NP. Nude mice bearing subcutaneous SK-N-MC xenografts in the flank were injected with ML111-NP containing the near-infrared dye SiNc. The NIR fluorescence signal of SiNc-loaded ML111-NP was detected in the xenograft as early as 1 h after single injection and reached maximum intensity between 6 and 10 h after injection (Figure 6D). A strong NIR fluorescence signal was observed in the xenograft, as compared to other organs collected at 24 h, which further confirmed accumulation and retention of ML111-NP within the xenograft (Figure 6D).

Mice bearing ~40–60 mm^3^ tumors (Figure 6E) were injected intravenously with ML111-NP at two doses, 4.5 and 15 mg/kg, every other day for 19 days. Two groups of control mice received equal volume injections of 5% dextrose or empty nanoparticles in 5% dextrose. A 67% and 85% reduction in tumor growth was observed in 4.5 and 15 mg/kg treatment groups, respectively, after 19 days (Figure 6F). Mice in both control groups were euthanized before 28 days due to the limitation of allowable tumor volume (2000 mm^3^), while ML111-NP treated group survived until day 48, at which point the mice were euthanized for further histological and toxicity studies.

Mice treated with ML111-NP did not exhibit loss of body weight (Figure S5), suggesting that ML111-NP was well tolerated and non-toxic over the course of these studies. Neither dose-limiting toxicity nor maximum tolerated dose could be determined due to the lack of toxicity of ML111-NP over the dosage range utilized in the present study.

Xenografts were sectioned and examined by immunohistochemistry for levels of p-H3^Ser10^, a marker of mitosis that was elevated in SK-N-MC cells treated with ML111 in vitro (see Figure 4B,D). Xenografts from ML111-NP-treated mice exhibited two-fold greater levels of p-H3^Ser10^ than those from control xenografts, consistent with a ML111-induced block in prometaphase of the cell cycle in vivo (Figure 6G and Figure S6).

Post-mortem analyses of treated and control mice also included the evaluation of ML111-induced toxicity to major organs by assessment of surrogate biomarkers and toxicity to the hematopoietic system by examination of circulating levels of various cellular components of blood and parameters of the circulatory system (Figure S7). None of these measurements were consistent with adverse effects of ML111 in vivo over the time-course of these studies.

## 4. Discussion

Ewing’s sarcoma patients with metastatic or relapsed disease have poor outcomes despite intense and aggressive multimodal treatment strategies, including high-dose chemotherapy regiments. Despite the identification of pathognomonic fusions involving EWSR1 and FLI1 genes nearly thirty years ago, there is a dearth of approved molecularly targeted therapeutic agents in clinical use. Notably, EWS-FLI1 and related fusions are deemed essentially undruggable because of the absence of intrinsic enzyme activity and a high degree of predicted disorder in the protein structure [41]. Intensification of preclinical discovery efforts has resulted in the identification of novel agents (e.g., TKI-216, IMG-7289) that are currently being investigated in clinical trials; however, the efficacy and, importantly, the durability of these agents are still unknown.

Therapeutic resistance is a nearly inevitable liability in the case of targeted therapeutics; thus, the development of multiple agents for use in the pharmacological arsenal is essential. Here, we undertook a high-throughput phenotypic screen to identify compounds with activity against Ewing’s sarcoma. We utilized a forward pharmacological screen, coupled with in silico mining of a large chemical library in order to identify new agents capable of inhibiting Ewing’s sarcoma cell viability. We postulated that a high-throughput phenotypic strategy would be advantageous in this case, given the challenges associated with identifying specific target proteins (for a reverse pharmacological approach) downstream of the complex, dysregulated transcriptional program driven by EWS-FLI1 or related fusion oncogenes. Furthermore, this target-agnostic approach has the potential to assist in the identification of previously unknown synthetic vulnerabilities that operate concomitantly or downstream of the fusion protein.

After identification of the lead compound, in silico mining resulted in the discovery of ML111. ML111 possesses nearly uniform potency again all six Ewing’s sarcoma cell lines tested, but it is inactive in many other cancer cell lines, including lung and breast carcinoma, as well as non-neoplastic cells such as HUVEC (Table S1). However, it is intriguing that ML111 potently inhibited the EML4-ALK fusion-driven H3122 and KRAS/PI3KCA-mutant H460 lung cancer cell lines. Since the oncogenic driver varies between the Ewing’s sarcoma and the ML111-sensitive lung cancer cell lines, we posit that a downstream vulnerability (such as a synthetic lethal interaction) may be the target of ML111. Future target deconvolution studies will take these findings into account and utilize proteomic as well transcriptomic approaches for identifying the ML111 target(s) in these cells.

Cumulative data from our cell cycle analyses (arrest at or near G2/M), microtubule polymerization assays, and biochemical assessment of cell cycle associated proteins (cyclin B1, phosphorylation of histone (H3), and CDC20) collectively demonstrate that ML111 induces M phase arrest that is independent of a direct effect on microtubule polymerization. Chromatin condensation is a requisite event during mitosis and precedes the alignment of sister chromatids to the metaphase plate and eventually controlled the symmetrical segregation of sister chromatids during anaphase. Phosphorylation of histone H3 on serine 10 is strongly correlated with chromosomal condensation during cell division [42]; in our studies, p-H3^Ser10^ antibody staining of ML111 treated cells revealed condensed but unaligned chromatin. Additional mechanistic insight into the role of ML111 comes from global proteomic studies. These data revealed the accumulation of proteins that are targeted for degradation by the APC/C ubiquitin ligase complex in cells treated with ML111. Future studies will interrogate whether ML111 perturbs enzymatic activity of the APC/C complex by directly binding to target protein(s) or disrupting key protein–protein interactions critical for the function of this large protein complex.

The current standard of care treatment for Ewing’s sarcoma relies on a multidisciplinary approach combining chemotherapy, surgery, and radiation therapy to control the primary tumors and limit metastasis [43]. Chemotherapy combinations include those involving vincristine (V), an inhibitor of microtubule polymerization; doxorubicin (D), a topoisomerase inhibitor; and cyclophosphamide (C), a DNA alkylating agent. The VDC treatment is alternatively cycled with ifosfamide, another DNA alkylating agent, and etoposide (a topoisomerase inhibitor) [44]. Several other combinatorial therapies for recurrent disease have been introduced, including gemcitabine plus docetaxel, irinotecan plus temozolomide [45], and topotecan and cyclophosphamide to optimize the balance between effectiveness and toxicity [43,46]. These chemotherapeutic regiments are largely non-selective and cytotoxic in nature, and despite intensive combination chemotherapeutic treatment approaches, the overall survival rates remain plateaued at ~75% for primary disease and less than 30% for metastatic disease [2,41,43].

Several active efforts for re-evaluating existing or investigate new anti-Ewing’s sarcoma agents are ongoing [3,47]. A promising new compound is TK216, a small molecule that binds to EWS-FLI1, and by disrupting its interaction with RNA helicase A, it suppresses the downstream oncogenic transcriptional program to induce apoptosis and reduces tumor burden in Ewing’s sarcoma cell and xenograft models [48]. TK216 is currently being evaluated as a single agent and in combination with vincristine in a phase I clinical trial (NCT02657005) for Ewing’s sarcoma patients with relapsed or refectory disease [48].

Inhibitors of Insulin-like growth factor 1 receptor (IGF1R) and poly-(ADP-ribose) polymerase (PARP1) are also under investigation for treatment of Ewing’s sarcoma [49,50]. Despite significant preclinical activity of an IGF1R inhibitor in Ewing’s sarcoma models, the inhibitor and anti-IGF1R monoclonal antibodies cixutumumab and ganitumab have demonstrated only modest or partial response in a subset of patients [51], suggesting that a biomarker to predict responsiveness to IGF1R inhibition may be required for patient selection. While single-agent treatment with olaparib, a PARP1 inhibitor, effectively inhibited Ewing’s sarcoma cell growth in vitro, monotherapy with olaparib failed to suppress Ewing’s sarcoma xenograft tumor growth or provide survival advantage in vivo [50,52] and showed no clinical benefit in a phase II trial with continuous high-dose treatment as a single agent [50]. However, the combination of olaparib with temozolomide or irinotecan showed significant responses in 80% of mice orthotopically engrafted with human Ewing’s sarcoma cells [52], and clinical trial data exploring combinations of PARP inhibitors in Ewing’s sarcoma are pending. In a separate study, combination treatment involving olaparib and radiation amplified DNA damage in preclinical model of Ewing’s sarcoma; however, the clinical benefits remain to be determined.

Nanoparticles have been extensively explored for the imaging and treatment of various cancers, including Ewing’s sarcoma [53,54,55,56]. Biocompatible and biodegradable PEG-PCL nanoparticles play a particularly significant role in biomedical engineering and drug delivery fields [57]. Numerous preclinical studies validated that PEG-PCL nanoparticles are safe and do not induce acute and chronic toxicity in animals following single or multiple injections [16,57,58]. Moreover, PEG-PCL nanoparticles significantly enhance drug accumulation at the cancer site following systemic administration [40,57]. Our in vivo results demonstrate that the PEG-PCL nanoparticles described herein efficiently accumulate in and are retained by Ewing’s sarcoma xenografts without the hallmarks of toxicity. Most importantly, our studies indicate that a nanoparticle-based ML111 formulation significantly inhibited SK-N-MC xenograft growth in vivo. Vincristine is one of the several chemotherapeutic drugs currently used to manage the disease (see above). In order to obtain the maximum therapeutic effect with minimal toxicity in the treatment of Ewing’s sarcoma, we recently showed that ML111 can combine synergistically with vincristine to lower the dose required to inhibit xenograft tumor growth [53]. Of note, previous reports validated that PEG-PCL nanoparticles can be loaded with multiple drugs, allowing concurrent delivery of potentially synergistic chemotherapeutic combinations to tumors [40]. PEG-PCL nanoparticles can also be functionalized with various ligands (e.g., peptides, antibodies, etc.) to target one or more therapies to a particular tumor type [57].

## 5. Conclusions

In summary, we have demonstrated that ML111 inhibits growth and induces apoptosis of Ewing’s sarcoma cells in vitro and suppresses growth of Ewing’s sarcoma tumors in a mouse xenograft model without apparent toxicity. ML111 induces a pro-metaphase arrest of Ewing’s sarcoma cells that may be associated with context-dependent inhibition of the APC/C complex, which harbors ubiquitin E3 ligase activity. We hypothesize that direct or indirect inhibition of the APC/C complex by ML111 results in the accumulation of multiple cell cycle proteins that must be degraded for progression through the G2/M checkpoint. Collectively, these actions result in enforcement of the spindle assembly checkpoint and initiation of an apoptotic response. The identification of the direct target of ML111 action should facilitate elucidation of the precise mechanism of ML111 action.

## Data Availability

Proteomics data have been deposited to the ProteomeXchange Consortium (http://proteomecentral.proteomexchange.org (accessed on 12 August 2021)) via the MassIVE partner repository with the dataset identifier PXD023150 and can be downloaded from the following link: ftp://massive.ucsd.edu/MSV000086599 (accessed on 12 August 2021).

## Supplementary Materials

The following are available online at https://www.mdpi.com/article/10.3390/pharmaceutics13101553/s1, Figure S1: ML111 induced apoptosis in Ewing’s sarcoma cell lines, Figure S2: Full-resolution cryo-TEM image of ML111-nanoparticles, Figure S3: Size distribution of ML111-NP as determined by dynamic light scattering, Figure S4: Fluorescence images of SK-N-MC cells treated with ML111-NP co-encapsulated with NIR SiNc dye, Figure S5: Effect of ML111-NP on total body mass, Figure S6: Quantification of immunohistochemical staining for p-H3^Ser10^ in sections of mouse xenograft, Figure S7: In vivo assessments of the acute toxicity of ML111-NP, Table S1: Effect of ML111 on viability of cancerous and primary cell lines, Table S2: Results of quantitative mass spectrometry analysis of the effect of ML111 on the SK-N-MC cell whole proteome, Table S3: Particle size (diameter), zeta potential, encapsulation efficacy, and loading capacity values of different polymer concentrations.

## Appendix A

**Table A1:** Quantification of proteins appearing in Figure 5A. Values are log2-fold relative downregulation (shades of blue) and upregulation (shades of red).

1.5	3	6	12	24 h	Protein	Uniprot Acc	Gene Symbol	APC/C Substrate References
Accumulating Cell Cycle Proteins								
0.29	−0.21	0.38	0.74	1.96	Borealin	Q53HL2	CDCA8	[59]
0.12	0.11	0.40	0.85	1.67	SGO2	Q562F6	SGO2	[60]
0.83	0.19	0.16	0.82	1.67	Histone H3.2	Q71DI3	HIST2H3A	[61]
0.32	−0.25	0.26	0.59	1.60	GTSE1	Q9NYZ3	GTSE1	[62]
0.27	−0.39	0.27	0.68	1.59	Survivin	O15392	BIRC5	[63]
0.34	0.02	0.33	0.59	1.56	TACC3	Q9Y6A5	TACC3	[64]
0.37	−0.18	0.33	0.63	1.53	NuSAP	Q9BXS6	NUSAP1	[65,66]
0.12	−0.04	0.23	0.68	1.47	INCENP	Q9NQS7	INCENP	
0.12	−0.04	0.26	0.47	1.43	UBE2S	Q16763	UBE2S	[67]
0.23	−0.20	0.42	0.46	1.42	Securin	O95997	PTTG1	[68,69]
0.13	−0.03	0.41	0.71	1.38	TPX2	Q9ULW0	TPX2	[70]
0.09	0.26	0.46	0.76	1.34	BUB1	O43683	BUB1	[71]
0.37	−0.29	0.15	0.34	1.22	ZWINT	O95229	ZWINT	[72]
0.23	−0.13	0.28	0.38	1.21	Tome−1	Q99618	CDCA3	[73]
0.81	0.34	0.36	0.75	1.16	LSM14B−var	A0A0C4DFV2	LSM14B	[60]
0.08	−0.05	0.15	0.33	1.12	HURP	Q15398	DLGAP5	[66]
0.25	0.18	0.39	0.53	1.10	HMMR	O75330	HMMR	[66]
0.05	−0.19	0.23	0.46	1.09	PIMREG	Q9BSJ6	PIMREG	[74]
0.08	0.00	0.13	0.43	1.05	Ki−67	P46013	MKI67	[75]
−0.26	0.41	0.52	0.83	1.00	Cyclin B1	P14635	CCNB1	[76]
0.06	0.14	0.31	0.51	1.00	PRC1	O43663	PRC1	[77,78,79]
0.02	0.15	0.35	0.57	0.97	CENP−E	P04183	TK1	[80]
−0.06	0.08	0.21	0.49	0.97	TK1	Q02224	CENPE	[81]
−0.11	0.15	0.30	0.55	0.96	Anillin	Q9NQW6	ANLN	[82]
−0.27	0.26	0.39	0.72	0.95	Aurora A	O14965	AURKA	[83]
−0.25	0.34	0.36	0.68	0.95	Aurora B	Q96GD4	AURKB	[84]
0.40	−0.01	0.24	0.66	0.94	Sororin	B5MBX0	CDCA5	[85,86]
0.00	0.15	0.32	0.51	0.91	CENP−F	P49454	CENPF	[81]
−0.09	0.24	0.37	0.57	0.89	KIFC1	Q9BW19	KIFC1	[87]
0.09	0.10	0.29	0.53	0.87	CKAP2	Q8WWK9	CKAP2	[88]
−0.07	−0.04	0.04	0.48	0.85	Repo−Man	Q69YH5	CDCA2	[89]
0.52	−0.33	0.38	0.50	0.84	Geminin	O75496	GMNN	[90]
0.16	−0.04	0.34	0.67	0.83	CDC20	Q12834	CDC20	[62]
−0.02	−0.04	0.22	0.35	0.83	Spindly	Q96EA4	SPDL1	
0.05	−0.24	0.14	0.24	0.82	UBE2C	O00762	UBE2C	[91,92]
0.04	0.19	0.32	0.57	0.81	KIF18A	Q99661	KIF2C	[87]
−0.20	0.13	0.24	0.46	0.81	KIF2C	Q8NI77	KIF18A	[93]
0.07	0.12	0.33	0.62	0.80	KIF22	Q14807	KIF22	[94]
−0.35	0.19	0.22	0.50	0.80	KIF20A	O95235	KIF20A	[74]
−0.24	0.35	0.34	0.60	0.73	PLK1	P53350	PLK1	[95]
−0.58	0.26	0.12	0.49	0.71	Top2α	P11388	TOP2A	[96]
0.45	−0.25	0.09	0.16	0.70	LSM14B	Q9BX40	LSM14B	[60]
0.49	−0.21	0.11	0.15	0.65	UPF3B	Q9BZI7	UPF3B	[60]
−0.38	0.19	0.20	0.48	0.59	ECT2	Q9H8V3	ECT2	[88,97]
Non−accumulating Prophase APC/C substrates								
0.28	0.06	0.29	−0.01	0.33	HOXC10	Q9NYD6	HOXC10	[36]
−0.18	−0.04	0.37	0.30	−0.49	Cyclin A2	P20248	CCNA2	[35]
