# Supplementary material for: Discovery and Validation of a Compound to Target Ewing’s Sarcoma
Source: Pharmaceutics. 2021 Sep 24;13(10):1553. doi: 10.3390/pharmaceutics13101553 (PMC8538197; doi:10.3390/pharmaceutics13101553)
Supplement: Supplementary file 1 [file pharmaceutics-13-01553-s001.zip › pharmaceutics-1359702 - supplementary.pdf]

pharmaceutics

Supplementary Materials: Discovery and validation of a compound to target Ewing's sarcoma

Ellie Esfandairi Nazzaro, Fahad Y. Sabei, Walter K. Vogel, Mohamad Nazari, Katelyn S. Nicholson, Philip R. Gafken, Olena Taratula, Oleh Taratula, Monika A. Davare and Mark Leid

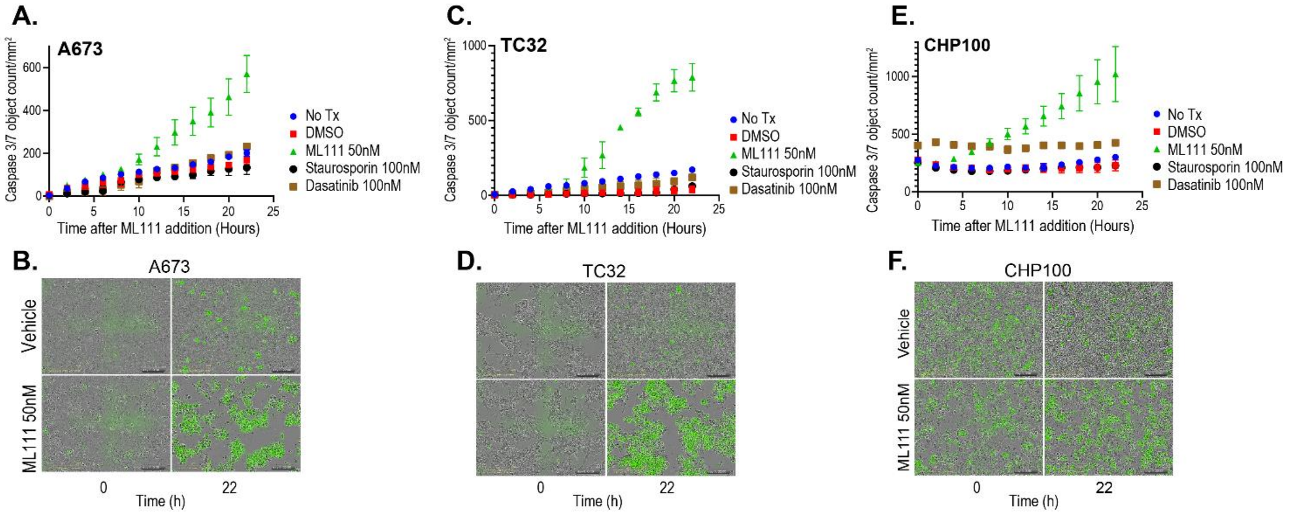

Figure S1. ML111 induced apoptosis in Ewing's sarcoma cell lines. Quantification of caspase 3/7 activation (apoptosis induction) and representative images from A673 (**A** and **B**) TC31 (**C** and **D**) and CHP100 (**E** and **F**) Ewing's sarcoma cell lines. Conditions: Untreated (blue circle), DMSO (vehicle; red square), 50 nM ML111 (green triangle), 100 nM staurosporine (black circles; positive control for death, multikinase inhibitor), and 100 nM dasatinib (brown squares; targeted multki-kinase inhibitor). After addition of indicated compounds, cells were live-imaged every 2 h for a total 22 h. Green fluorescence indicates cellular caspase 3/7 activation as detected using CellEvent™ Caspase-3/7 Green Detection Reagent. The graphs show average ± SEM (n = 3). Scale bar in panels B, D and F represents 200 µm.

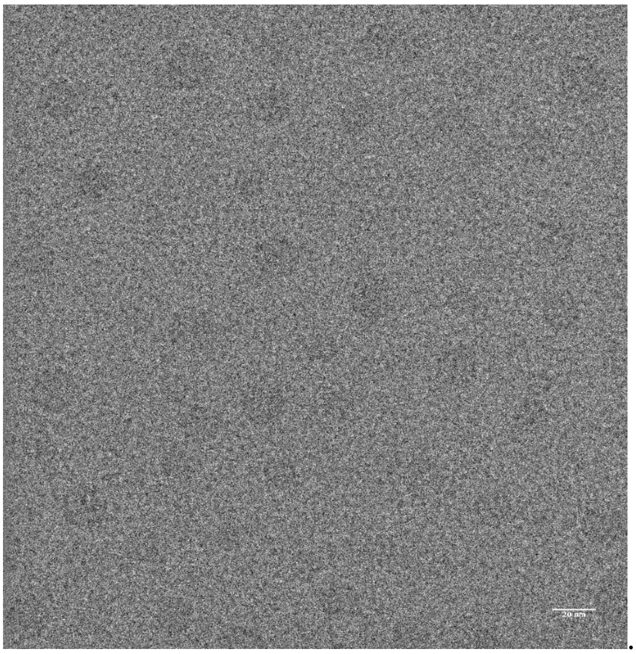

Figure S2. Full-resolution cryo-TEM image of ML111-nanoparticles (16-bit grayscale). Scale bar = 20 nm.

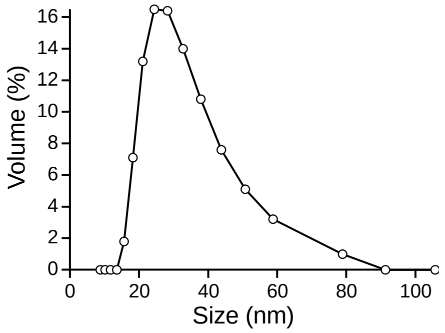

Figure S3. Size distribution of ML111-NP as determined by dynamic light scattering. ML111-NPs have a mean hydrodynamic diameter of 31.8 ± 0.2 nm.

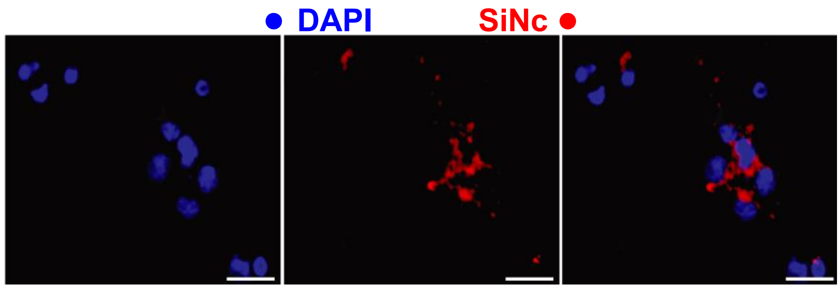

Figure S4. Fluorescence images of SK-N-MC cells treated with ML111-NP co-encapsulated with NIR SiNc dye (2 μg/mL, red) for visualization. Cell nuclei were counterstained with DAPI (blue). Magnification 20×. Scale bar = 100 μm.

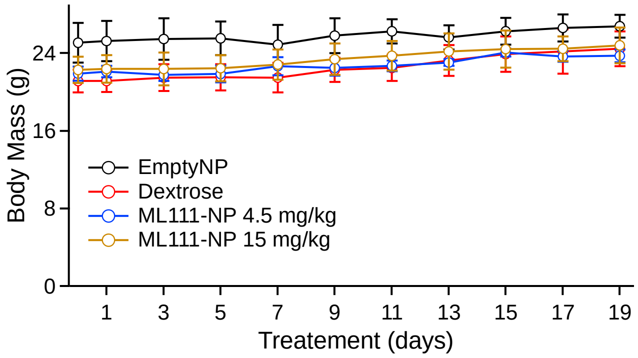

Figure S5. Effect of ML111-NP on total body mass. Lack of body mass change of mice during the treatment course with ML111, indicating lack of ML111 toxicity. Data are shown as means ± SD; (n = 5).

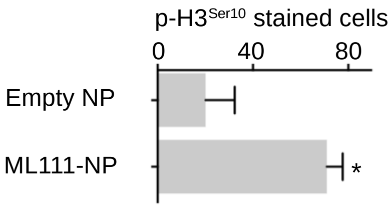

Figure S6. Quantification of immunohistochemical staining for p-H3^{Ser10} in sections of mouse xenograft. Data represented as means of multiple fields ± SD.

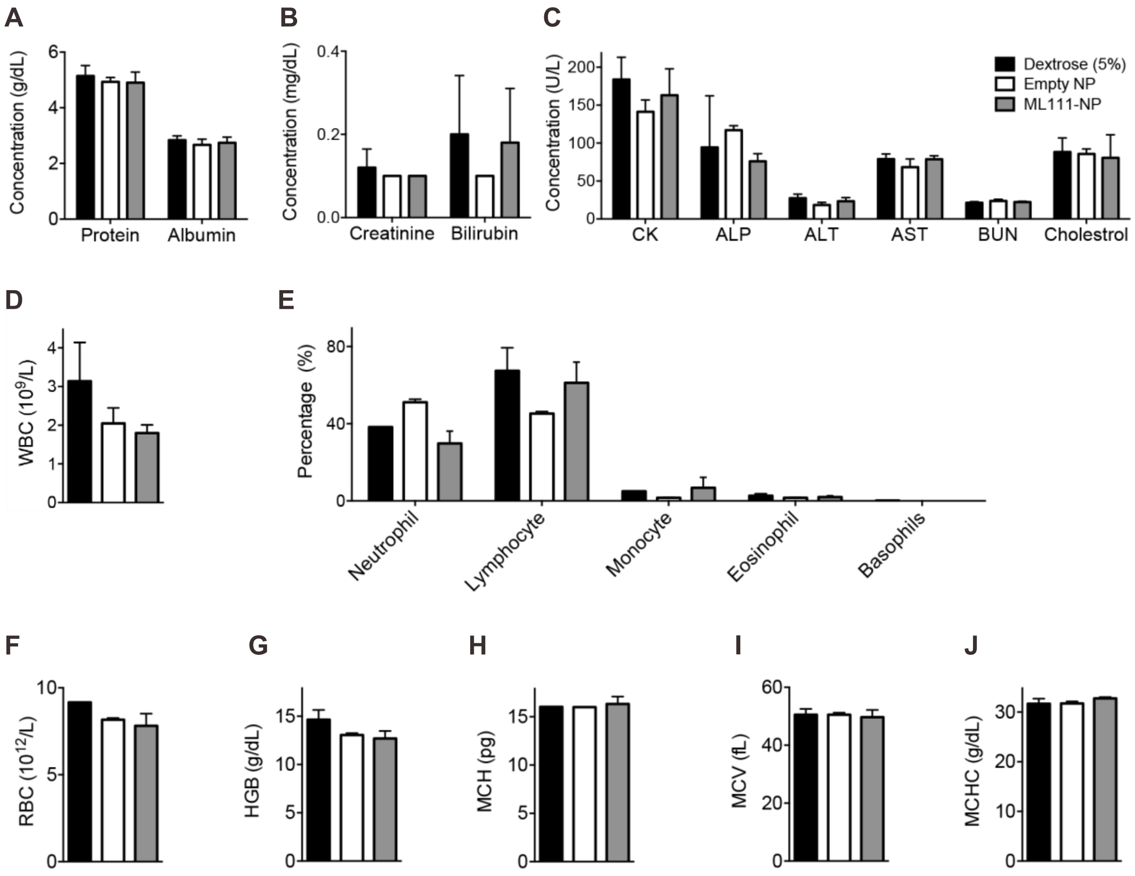

Figure S7. In vivo assessments of the acute toxicity of ML111-NP. Blood panel (hepatic, renal and blood factors) after i.v. injection of dextrose 5% (black), empty-NP (white) and ML111-NP at 15 mg/kg dose three times per week for three consecutive weeks (gray; $n = 5$). Significant differences were not observed in hepatic, renal and blood parameters amongst the treatment groups. Abbreviations: ALP, alkaline phosphatase; ALT, alanine transaminase; AST, aspartate amino-transferase; BUN, blood urea nitrogen; CK, creatine kinase; HGB, hemoglobin; MCH, mean corpuscular hemoglobin; MCHC, mean corpuscular hemoglobin concentration; MCV, mean corpuscular volume; RBC, red blood cell; WBC, white blood cell.

Table S1. Effect of ML111 on viability of cancerous and primary cell lines. IC_{50} values of ML111 in cancerous and primary cell lines. ML111 selectively induced cell death in cancer cells and showed less potency in human primary cell lines.

Cell line	Cell type	IC_{50} ± SD (nM)
SK-N-MC	EwS, Type I EWS/FLI-1	21.4 ± 1.4
A-673	EwS, Type I EWS/FLI-1	38.37 ± 2.11
TC-71	EwS, Type I EWS/FLI-1	38.38 ± 1.24
CHP100	EwS, Type II EWS/FLI-1	29.70 ± 3.71
TC-32	EwS, Type II EWS/FLI-1	33.85 ± 1.8
SK-ES-1	EwS, Type II EWS/FLI-1	21.83 ± 3.21
MDA-MB-231	Breast cancer	>1000
HCC78	Non-small cell lung carcinoma	>1000
SK-OV-3	Ovarian cancer	>1000
HEK293	human embryonic kidney cells 293	>1000
HUVEC	Human umbilical vein endothelial cells	>1000
H460	Lung Cancer	33.3 ± 7.4
ES-2	Ovarian cancer	27.07 ± 1.12
H3122	Non-small cell lung carcinoma	9.84 ± 0.85

Table S2 (separate file). Results of quantitative mass spectrometry analysis of the effect of ML111 on the SK-N-MC cell whole proteome. Protein identifications, confidences, and normalized protein abundances for 7122 proteins are shown.

Table S3. Particle size (diameter), zeta potential, encapsulation efficacy, and loading capacity values of different polymer concentrations.

NP	ML111 (mg/mL)	Polymer (mPEG-PCL) (mg/mL)	Mean size ± SD (nm)	PDI ± SD	Mean zeta potential (ζ) ± SD (mV)	Entrapted drug (mg/mL)	EE%	LC%
Empty NP	–	80	31.5 ± 0.52	0.10 ± 0.02	–	–	–	–
ML111-NP-20	2.25	20	35.3 ± 0.12	0.06 ± 0.09	-0.69 ± 0.02	0.34	15.1	1.52
ML111-NP-40	2.25	40	35.5 ± 0.13	0.10 ± 0.08	-1.05 ± 0.03	1.51	67.1	3.57
NP111-NP-60	2.25	60	32.6 ± 0.14	0.08 ± 0.09	0.18 ± 0.08	1.70	75.5	2.74
ML111-NP-80	2.25	80	31.8 ± 0.19	0.070 ± 0.09	0.12 ± 0.01	2.01	89.3	2.44
